# Matching Qualitative Inquiry Design and Practice to Contemporary Burns Research Questions: Are We Getting It Right?

**DOI:** 10.3390/ebj3020022

**Published:** 2022-03-28

**Authors:** Megan Simons, Jodie A. Copley

**Affiliations:** 1 Centre for Children’s Burns and Trauma Research, Child Health Research Centre, The University of Queensland, 62 Graham Street, South Brisbane, QLD 4101, Australia; 2 Occupational Therapy Department, Queensland Children’s Hospital, 501 Stanley Street, South Brisbane, QLD 4101, Australia; 3 School of Health and Rehabilitation Sciences, The University of Queensland, Brisbane, QLD 4067, Australia; j.copley@uq.edu.au

**Keywords:** burns, cicatrix, scar, qualitative methodology, post-qualitative

## Abstract

Qualitative methodology has continued to develop through scholarly inquiry, with its application to burn scar research progressed substantially since early use. Concerns were raised in 2015 that qualitative inquiry in burn care and rehabilitation used a limited range of qualitative research approaches. The aim of this commentary paper is to consider how broadly the suite of methodologies available within the qualitative research paradigm have been applied to burn scar research since that call. Observations from a scan of qualitative burn scar papers published since 2015 to March 2022 (*n* = 36) are presented. Less commonly used qualitative methodologies (such as interpretive design, interpretive phenomenological analysis, narrative inquiry, grounded theory, explanatory case study) and their contribution to burn scar research is discussed. Examples are presented to consider how the application of qualitative methodological approaches (including post-qualitative research methodologies) can be ultimately used to inform meaningful outcomes.

## 1. Background

Over recent decades, the application of qualitative methodology has become more integral to burns research resulting in, for example, awareness of the distance between what experts and people with burn scars considered important for scar outcomes [1,2]. Qualitative methodology is identifiable by its intention to inquire into, document, and interpret how we make meaning of our experiences, and how this interacts with our behaviours, attitudes, values and beliefs, goals and cultural perspectives [3]. Qualitative methodology has continued to develop through scholarly inquiry over several decades and is ideally suited to generate (or test) theory and contribute to knowledge building through a deep and rich understanding of the phenomenon under investigation [3]. Applying qualitative methodologies to understand the experience of burn scarring and burn scar interventions has progressed substantially since their early use in burns research [4,5]. Yet, the question remains as to whether we are getting the application of this methodology right.

Kornhaber et al.’s (2015) [6] integrative review showed that qualitative research methodologies in burn care and rehabilitation from 2009 to April 2014 predominantly focused on exploring experiences from the perspective of burn survivors and their families. These experiences ranged across the age continuum and continuum of care, in relation to return to work/school and with respect to the physical, psychosocial, and environmental aspects of burns trauma (including scarring). To generate this data, an overwhelming majority of studies used a semi-structured interview approach, with a limited range of other qualitative research alternatives such as observation, field notes or document review (including diaries, medical records, web sites) evident. Kornhaber et al. [6] also found limited description of any philosophical underpinnings of the research designs [7] used. Being aware of, and articulating, such underpinnings is often considered important to inform the framing of the research question and approach to analysis [3]. Its absence in burns studies suggests that qualitative methodological training in burns researchers was limited.

Over half of the papers in Kornhaber and colleague’s review [6] were generated from nursing, followed by allied health. Medical authorship was under-represented, suggesting that academic medicine was yet to embrace qualitative approaches. Kornhaber and colleagues argued for a more interprofessional approach to research in this field, to facilitate healthcare researchers to build the evidence base alongside patient and carer participation. More broadly, they called for rigorous assessment and evaluation of outcomes and services using qualitative and mixed research methods that respect the individual patient, their cultural context, and lived experience (of both the person with burns, their care providers and healthcare professionals). Seven years later, this commentary paper aims to consider how broadly the suite of methodologies available within the qualitative research paradigm have been applied to burn scar research since that call.

First, we will discuss our observations from a scan (rather than a formal scoping review) of qualitative burns scar papers published since 2015. Second, we will consider the (to date) less commonly used qualitative methodologies and what they have contributed to burn scar research. Finally, we will consider how the application of qualitative methodological approaches in a more informed way can be used to frame the data collection and analysis methods. Recent developments in qualitative methodologies will be described, including post-qualitative methodology, with consideration of its potential to progress burn scar research.

## 2. Where Are We Now?

Two databases (EMBASE, PubMed) were searched from January 2015 to March 2022 (see Supplementary File S1 for search strategy). Abstracts were screened (by MS and JC) for original qualitative burn scar research. Reference lists of retrieved articles were also scanned by MS. The retrieved full-text articles (*n* = 36) were read by MS to extract epistemology, participant details, objective/s of the study, analysis techniques, findings, and qualitative credibility and trustworthiness strategies.

So, what progress has been made in the last seven years? From the literature scan, the qualitative approaches used in burn scar research during this period have broadened to include qualitative description (12 studies) [8,9,10,11,12,13,14,15,16,17,18,19]; interpretive designs (8 studies) [20,21,22,23,24,25,26,27]; phenomenology (8 studies) [28,29,30,31,32,33,34,35], mixed methods (2 studies) [36,37], systematic reviews of qualitative studies (2 studies) [2,38], grounded theory (3 studies) [39,40,41] and ethnography (1 study) [42]. This scan of a range of qualitative burns papers from 2015–March 2022 suggests that qualitative inquiry has contributed greatly and expanded our understanding of the patient’s (or their caregiver’s) perceptions of burn scarring [28], their experiences of living with an altered appearance [8,9,16,18,20,29,30,31,39] and using scar interventions (such as pressure garments) [15,21,22,23,24,32,36]. Our understanding of the impact of culture on one’s lived experience of scarring [9,18], and important items to include in patient-reported outcome measures of burn-scar health-related quality of life [2,10,25,37,41] have both been more richly informed using a qualitative approach. Qualitative inquiry has also deepened our understanding of the experience of the health professionals who provide psychosocial services [11,26,27] and supportive interventions that provide relief to people with burn scars [13,14,19,33]. The importance of these findings to clinical care lies in a deeper understanding of how biopsychosocial issues impact recovery and ability to advocate for meaningful outcomes to people with burn scars.

In recent years, the qualitative data gathering method of choice has remained semi-structured interviews or focus groups, using purposive sampling of adults or parents of children with burn injuries (proxy reporting) [8,9,10,11,20,24,25,26,27], convenience sampling [15,18,19,28,29,37,39] or recruitment until a pre-specified time-period lapsed [16,23,31,32]. Despite the variety of research designs, for which a range of analysis types might be suited (for example, framework analysis [43] or interpretive thematic analysis [44]), most analyses in these studies were undertaken using qualitative content analysis [45] or thematic analysis [46]. What is interesting is that these preferences continue to dominate, despite the diversity of the research questions investigated in these studies. Whilst it is possible that semi-structured interviews and content/thematic analysis continue to be most appropriate, given the broader range of research questions involved in these studies, and the potential research questions we could ask that are appropriately answered using qualitative methodology, it is likely that these ongoing preferences are due to limited experience and understanding of how the range of qualitative methods can be applied.

A further question to consider is how the trustworthiness and credibility [7] of burns qualitative research has progressed over the past seven years. Qualitative ‘technical fixes’ [47] included in checklists such as Consolidated Criteria for Reporting Qualitative Research (COREQ) and Standards for Reporting Qualitative Research (SPQR) [48,49] strengthen the rigour of qualitative research when embedded in a broader understanding informing the alignment of research design and data analysis, as opposed to being applied in a prescriptive way. Signs of prescriptive application from the scan of recent qualitative papers specific to burn scar interventions included use of purposive sampling without considerations of desired spread of participant characteristics. Whilst some studies may be seeking consensus among participants, others may want to capture diversity of experience to inform service provision. In the latter case, it may be important to use a matrix to sample sufficient numbers of participants in a targeted way who are known to have, for example, different personal circumstances, burn characteristics and geographical locations. In that type of study, the concept of reaching data saturation (commonly mentioned in burns qualitative papers) is not relevant. Another potential sign of prescriptive application was that multiple coders were frequently used, often for the demonstration of coding consensus. However, the value of different perspectives when coding lies in the disagreements to alert researchers to potentially competing explanations, with a traceable audit trail of the process used to de-mystify the emergence of interpretation of the data [3,7]. Member checking [50] is often used in burns qualitative research, but this is generally through the return of interview transcripts to participants (i.e., raw data), rather than provision of preliminary interpretations of the data for participant checking and input, which has more potential to improve credibility of the findings. However, the emergence of qualitative papers with thick description addressing reflexivity [26] and linkage of philosophical basis to data interpretation [8,23,26] are positive signs that burns researchers are broadening their understanding of qualitative research methodology.

## 3. The Road Less Travelled

Qualitative frameworks less frequently seen in burns research (such as grounded theory, narrative inquiry, phenomenology, and more) represent varied philosophical and theoretical perspectives, each of which informs research design and offers an alternative approach within the qualitative paradigm [3]. One of the novel qualitative methodologies (grounded theory) applied since 2010 in burns research has informed theory grounded in people’s experiences of surviving burn injury within an Indian cultural context [51,52], resulting in a deeper understanding of the four stages of ‘Enduring the Blame’ from family members, health professionals, strangers, and their child with burn scars. Another used analysis of storytelling to deepen our understanding of the impact of the societal context on men’s recovery post-burn [12]. Explanatory case design was applied to predict (upon admission) paediatric burn outcomes at 6-months post-burn in school-aged children [53]. The inclusion of family and social functioning in the resulting conceptual model has been further supported in subsequent qualitative and mixed-method studies [10,41]. Interpretive designs have informed clinical practice through a deeper understanding of the lived experience of adults using pressure garments as scar interventions, such as how treatments are integrated into self-identity, body image and routines and the psychosocial features of these interventions (such as relying on the perceived protection offered by the pressure garment) [23]. Such new learnings from contemporary qualitative inquiry inform health professionals about the individual nature of burn scar experiences, and by doing so help tailor the support provided and potentially improve adherence to treatment [21,24], ultimately supporting meaningful outcomes.

## 4. Where to from Here?

To optimise the return on this resource-heavy qualitative research investment, the most useful next step might be returning to the foundations of our research questions themselves, and carefully considering what type of qualitative approaches will best answer them. For example, in clinical work it is often observed that a ‘small’ burn scar, with no discernible functional impact, will for some children be a source of persistent teasing and bullying at school. If the research question was ‘What is the nature or essence of the teasing and bullying experience after sustaining a burn scar (so the clinician can now better understand what this experience is like for children with burn scars to inform their clinical practice)’, then an interpretive orientation approach that acknowledges the individualism of the constructed and contextual nature of one’s experiences, whilst allowing for shared realities, is appropriate. Interpretive description [54,55] would be an option (as would narrative inquiry [56] or interpretive phenomenological analysis (IPA) [57]), where one could seek in-depth interviews, informed by a theoretical sampling of children (experiencing/who have experienced bullying and teasing and those who did not), their caregivers and (ideally) school staff. Meaning could be interpreted from their lived experiences using reflexive thematic analysis [58]. However, if the research question was to generate a theory or model about why some young people are negatively emotionally impacted by their burn scarring (such as teasing, bullying) to the point of impacting their engagement with everyday activities, one might lean towards using Grounded Theory Methodology [59] (based on a realism framework), which emphasises that truth is context dependent [3]. It would be appropriate to purposefully recruit school-aged children with ‘small’ burn scars (with functional and non-functional impact) for qualitative interviews. The constant comparative method of analysis could be used to generate (and test) theory about which factors explained the difference (impact of burn scarring on life engagement) [59]. While the current example focuses on interviews as a primary method of data collection, as this is appropriate to the research context, other questions about lived experience might prompt the researcher to consider diverse data collection methods, such as researcher field notes [3], photo elicitation [60] or observation followed by stimulated recall interviews to understand participant actions [61].

Given the plethora of individual and interdisciplinary understandings of qualitative terminology, burns clinical researchers may find benefit in (re)learning qualitative research methods alongside their academic qualitative research partners. The study of a person’s unique experience underpins the qualitative methodological approach. So, by its very nature, our goal as burns clinicians and/or burns researchers of supporting human thriving (and what constitutes human thriving) post-burn scar cannot be known in advance for the individual, through generalising research findings (or clinical experience). Embracing this position (as a clinician and researcher) is challenging for healthcare and funding systems that are more familiar with dominant research paradigms informed by positivist frameworks (e.g., examining assertions and corroborating claims) [62]. One emerging viewpoint is to reframe scientific inquiry as understanding generalisable knowledge, not as knowledge of stable objects of investigation but as knowledge about rapidly changing phenomena [63,64]. This represents a (re)positioning epistemologically in response to the quantification of qualitative approaches over time [54]. Post-qualitative research methodologies [63], whilst yet to emerge in burns research, have the potential to offer a fresh take on (for example) the question of “what is ‘person-centred’ burn scar rehabilitation care?” [64]. By re-aligning focus from individuals and human subjects to how relational networks/collections of animate and inanimate (for example, patient, practitioner, theatre setting, wound dressings, scar treatments) affect and are affected, burns researchers can contribute to ongoing efforts to understand, and improve, the experience of burn scar rehabilitation in ways yet unrecognised.

## 5. Conclusions

In conclusion, it is evident from even a scan of qualitative burn scar papers published after Kornhaber’s et al. (2015) [6] integrative review that qualitative inquiry has extended beyond exploring the experience of burn survivors and their families. There is increased use of mixed methods designs, with interprofessional representation (including medical authorship) as Kornhaber had implored. Qualitative methodological support for burns researchers appears to be improving, with more consistent description of the philosophical underpinnings used to frame the research question/s and approach to analysis. However, the potential for application of qualitative inquiry across the spectrum of burn scar research is yet to be fully realised. It is arguable that more burns scar research is needed that provides information to optimise (or help reconfigure) the translation of person-centred practice, patient experience and meaningful outcomes into clinical settings. Whilst the uptake of new qualitative methodologies is likely to take time (reflecting, for example, the uptake of evidence-based practice), particularly in a research system designed for randomised controlled trials (RCTs), the increasing depth and breadth of research findings generated by well-designed qualitative burn scar studies suggests that that time may just have arrived.

## Data Availability

The author M.S. can be contacted at megan.simons@health.qld.gov.au for information regarding access to the dataset.

## Supplementary Materials

The following supporting information can be downloaded at: https://www.mdpi.com/article/10.3390/ebj3020022/s1, Supplementary File S1: Search Strategy.
