# Supplementary material for: Matching Qualitative Inquiry Design and Practice to Contemporary Burns Research Questions: Are We Getting It Right?
Source: Eur Burn J. 2022 Mar 28;3(2):256–63. doi: 10.3390/ebj3020022 (PMC11575374; doi:10.3390/ebj3020022)
Supplement: Supplementary file 1 [file ebj-03-00022-s001.zip › ebj-1606873-supplementary.pdf]

Supplemental File 1 Search Strategy

("burn scar"/de OR ("burn scar*" OR "burns scar*"):ti,ab)
AND
(((semi-structured OR semistructured OR unstructured OR informal OR in-depth OR indepth OR face-to-face OR structured OR guide) NEAR/3 (interview* OR discussion* OR questionnaire*)):ti,ab
OR ("interpretive descript*" OR "case stud*" OR "focus group*" OR qualitative OR ethnograph* OR fieldwork OR "field work" OR "key informant"):ti,ab,kw OR "qualitative research"/de)
